# Structural basis of condensin II activation

**DOI:** 10.21203/rs.3.rs-9335492/v1

**Published:** 2026-06-05

**Authors:** Pierre-Jean Matteï, Dong-Hua Chen, David A. Bushnell, Baldandorj Baatar, Andrew J. Beel

**Affiliations:** 1 Department of Structural Biology, Stanford University, Stanford, CA 94305, USA

## Abstract

Condensin II is present in the nucleus throughout interphase, yet does not acquire robust loop-extruding activity until cells enter prophase. How the complex is kept inactive before prophase has remained unclear. Here we show, by cryo-EM and mass photometry, that nucleotide-free human condensin II forms a reversible dimer at physiological ionic strength. In this dimer, CAP-H2 elements required for chromosome association are buried, while the C-terminal tail of CAP-D3 holds the neck gate open by blocking engagement of the CAP-H2 neck-binding domain with the SMC2 neck. Nucleotide binding promotes monomerization and causes the SMC module to rotate toward the displaced neck-binding domain, but persistent CAP-D3 blockade keeps the gate open. These structures explain CAP-D3-dependent self-suppression of condensin II activity and suggest how nucleotide binding and M18BP1 engagement promote progression from an autoinhibited state toward a poised, pre-clamping intermediate.

## Introduction

As eukaryotic cells enter mitosis, chromatin is restructured from its dispersed interphase configuration into rod-shaped chromatids through an ATP-dependent process of “loop extrusion” that is carried out by members of the structural maintenance of chromosomes (SMC) family known as condensins [1, 2]. The catalytic activity of the condensins resides in a pair of coiled-coil ATPases — SMC2 and SMC4 — which assemble into a V-shaped heterodimer characterized by an apical “hinge” domain, situated at one end of the assembly, and a pair of ABC-type ATPase head domains at the other [3–5]. A substantially unstructured kleisin subunit contacts the SMC2 head-proximal coiled coil, or neck, through its N-terminal neck-binding domain (NBD), and the distal surface of the SMC4 head through its C-terminal winged-helix domain (cWHD) [6]; these interactions convert the V-shaped SMC heterodimer into a ternary SMC-kleisin ring structure that can topologically entrap DNA [7]. The kleisin also presents binding sites for two additional regulatory subunits that are rich in HEAT repeats [8, 9] and are therefore known as HAWKs (HEAT-repeat proteins associated with kleisins) [10].

The SMC2 neck and the kleisin NBD can associate to form the “neck gate” [11, 12], an important locus of control for the entrapment and release of DNA by SMC-kleisin complexes [13, 14]. In cohesin, a related complex that organizes the interphase genome and underlies sister-chromatid cohesion, the analogous Smc3-kleisin interface functions as a DNA-exit gate that controls the release of DNA from the SMC-kleisin ring [13, 15, 16]. Structures of yeast condensin have shown that nucleotide and DNA occupancy regulate the neck gate. In the apo state, and in the presence of both DNA and a nonhydrolyzable ATP analogue, the kleisin NBD forms a three-helix bundle with the two helices of the SMC2 neck, resulting in a closed neck gate [11, 12]. By contrast, when a nonhydrolyzable ATP analogue is bound to the SMC head domains in the absence of DNA, the neck gate opens but the kleisin NBD is not resolved, consistent with putative flexibility of the open state [11, 12].

Many eukaryotes, including humans, express two types of condensins, which differ in their regulatory subunits and patterns of subcellular localization [17]. One type, condensin I, is universal, whereas the other, condensin II, has been recurrently lost across the course of eukaryote evolution, and its absence or presence correlates with a Rabl-like or chromosome-territorial interphase genome architecture, respectively [18]. The kleisin subunit is alternately CAP-H (for condensin I) or CAP-H2 (for condensin II), and the HAWK subunits are alternately CAP-D2 and CAP-G (for condensin I) or CAP-D3 and CAP-G2 (for condensin II). Whereas condensin I is primarily extranuclear and therefore excluded from chromatin until nuclear envelope breakdown, condensin II is predominantly intranuclear and begins reshaping chromatin during early prophase [19, 20].

Despite its intranuclear residence, condensin II does not stably associate with chromatin during interphase [21, 22], and its loop-extruding activity during that period is restricted by the combination of an intrinsic, CAP-D3-dependent autoinhibitory mechanism [23, 24] and an extrinsic, MCPH1/M18BP1 switch [22, 25], both of which are subject to regulation by Cdk1 at mitotic entry. The autoinhibitory mechanism involves the C terminus of CAP-D3 (one of two HEAT-repeat subunits of condensin II), which contains an α-helical “HEAT docker” that engages the other HEAT-repeat subunit, CAP-G2 [24, 25], as well as a region rich in Cdk1 consensus sites that is predicted to be disordered [24]. Together, these features limit the capacity of condensin II to stably associate with chromatin, leading to a self-suppressed state that is thought to be relieved by Cdk1 phosphorylation [23, 24], consistent with the importance of Cdk1-mediated phosphorylation of CAP-D3 for the onset of chromosome condensation in prophase [26]. The extrinsic regulatory mechanism involves the binding of MCPH1 [22] and M18BP1 [25] to CAP-G2, and their phosphorylation-dependent exchange, which enables stable chromosomal loading of condensin II at mitotic entry [25].

Although biochemical and cellular studies have identified CAP-D3-dependent self-suppression [24] and M18BP1-dependent activation [25] as important elements of condensin II regulation, the structural basis for these mechanisms has remained unclear. To address this gap, we determined single-particle cryo-EM structures of human condensin II in nucleotide-free and nucleotide-bound states. These structures reveal the architecture of an autoinhibited condensin II complex in which the directly resolved CAP-D3 C-terminal tail sequesters the CAP-H2 NBD, providing a molecular explanation for the CAP-D3-dependent self-suppressive mechanism identified by Yoshida *et al*. [24]. Together with the published structure of M18BP1-bound condensin II [25], they further show how M18BP1 remodels the complex to promote an activated state.

## Results

**Figure 1:**
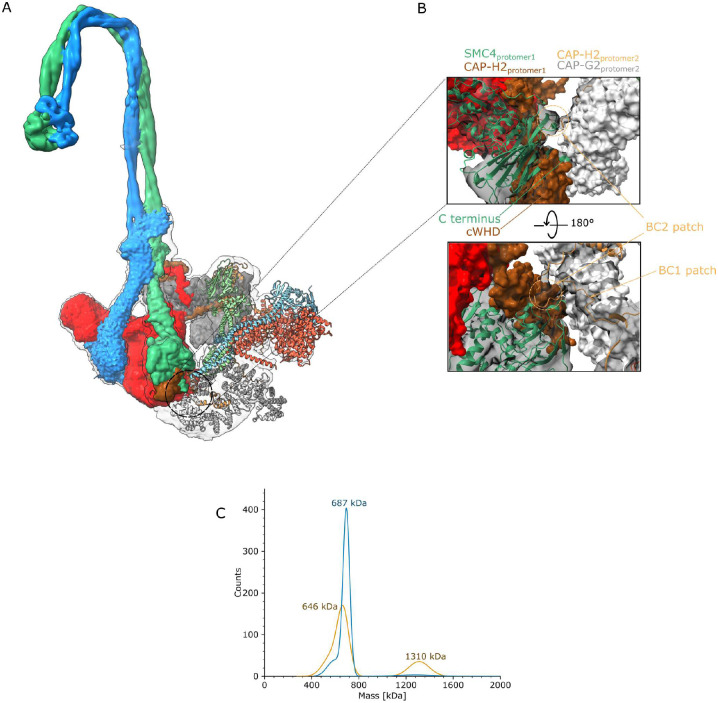
Human condensin II forms a dimer in solution. **(A)** Composite cryo-EM map with one protomer represented by EM density and the other by a cartoon model. Superposed in transparent gray is the total density of the dimer. Subunits are colored as follows: SMC2, blue; SMC4, green; CAP-H2, brown; CAP-D3, red; CAP-G2, gray. **(B)** Detailed view of the condensin II dimerization interface, formed by the SMC4 head domain and CAP-H2 cWHD of protomer 1 together with CAP-G2 HEAT repeats 11–13 of protomer 2. The interface buries the CAP-H2 BC2 segment of protomer 2. **(C)** Condensin II mass distributions in the presence of 300 mM KCl (blue) and 150 mM KCl (orange). Modal masses are indicated.

To better understand the regulatory mechanisms governing condensin II activity, we expressed and purified the human condensin II pentamer (Fig. S1) and determined the structure of the apoenzyme to 3.6 Å resolution by single-particle cryo-EM analysis (Fig. S2). The reconstruction unexpectedly revealed a large annular assembly (Fig. S2E), the dimensions of which could not be explained by a single SMC-kleisin ring. Model building showed that this density is instead accounted for by two condensin II molecules, arranged as a dimer of pentamers (Fig. 1A) with overall pseudo-C2 symmetry (Fig. S3). Such an arrangement has not been observed in experimental structures of other eukaryotic SMC complexes reported to date [11, 12, 27, 28]. The two protomers were not equally well resolved: focused refinement of the better-resolved protomer (Fig. S4) revealed continuous density for the full extent of the SMC2–SMC4 coiled coils and the hinge domain (Fig. S5), whereas the second protomer retained density only for the head-proximal coiled coils. We therefore generated a composite map by combining the overall dimer reconstruction with the focused SMC2–SMC4 coiled-coil/hinge map from the better-resolved protomer, providing a nearly complete view of the condensin II decamer (Fig. S3).

**Figure 2:**
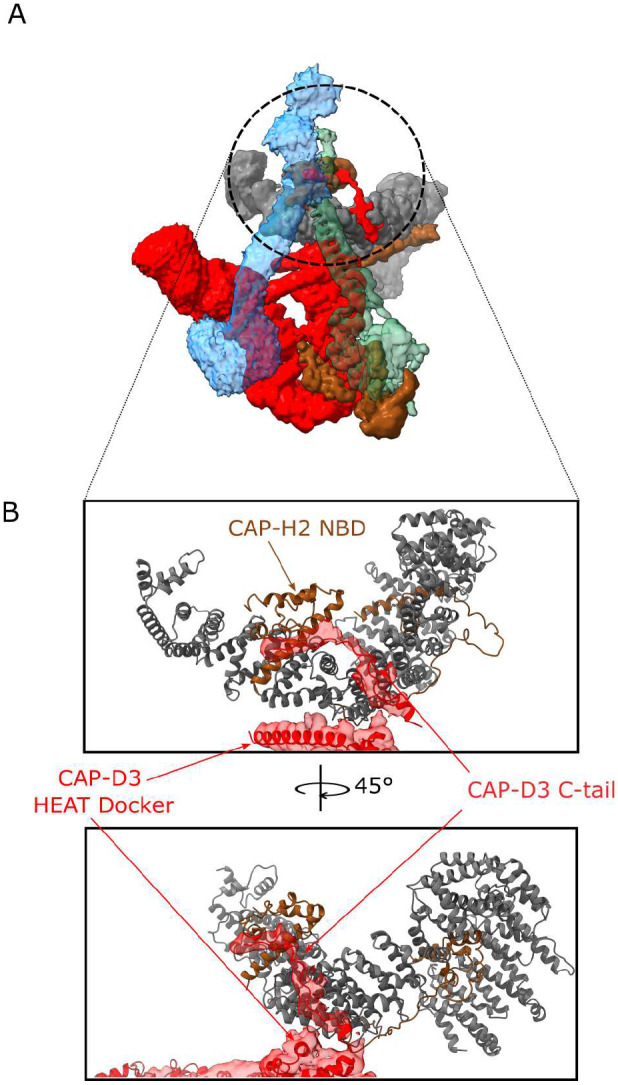
The CAP-H2 neck-binding domain is sequestered by the C-terminal tail of CAP-D3. **(A)** LocScale-sharpened cryo-EM map of protomer 1 with SMCs rendered transparently to reveal the CAP-G2/CAP-H2/CAP-D3 interface. **(B)** The CAP-H2 NBD (brown) is displaced from the SMC2 neck and sandwiched between the CAP-G2 body (gray) and the CAP-D3 C-terminal tail (red). The CAP-D3 “HEAT docker” α-helix anchors CAP-G2 to the SMC2–SMC4–CAP-D3 head module.

Within each protomer, the composite map reveals a conserved SMC2–SMC4–CAP-D3 head module (Fig. 1A) that resembles the SMC-kleisin core of yeast condensin [11, 12]. The SMC heads (blue and green density in Fig. 1A) are disengaged and separated by 3.5 nm, compared with 2 nm in the non-engaged state and 10 nm in the bridged state of yeast condensin [11]. A large density situated behind the SMC head domains was assigned to CAP-D3 (depicted in red in Fig. 1A); similar to Ycs4 (CAP-D2) in the non-engaged conformation of yeast condensin [6, 11], CAP-D3 predominantly contacts the SMC4 head, including an interaction between the highly conserved KG loop of CAP-D3 (K1014, G1015) and the W loop of SMC4 (W1185). In contrast to apo structures of yeast condensin, where Ycg1 (CAP-G) is not resolved due to its flexibly tethered association with the SMC–kleisin–CAP-D2 head module [11, 12], CAP-G2 is well resolved in human condensin II and is anchored to CAP-D3 through the latter’s α-helical “HEAT docker” motif (residues D1245–A1280; Fig. 2), consistent with recent predictive [24] and structural [25] models. We resolved 376 of 605 residues (62.1%) of CAP-H2 (brown density in Fig. 1A and Fig. 2), exceeding the kleisin coverage of prior condensin structures: 216/754 Brn1 (CAP-H) residues in apo non-engaged yeast condensin (28.6%; PDB 6YVU) and 207/754 in apo bridged yeast condensin (27.5%; PDB 6YVV) [11]; 279/754 in clamped yeast condensin–DNA (37.0%; PDB 7Q2X) [12]; and 252/605 CAP-H2 residues in human condensin II–M18BP1 (41.7%; PDB 9F5W) [25]. The resolved regions of CAP-H2 (Fig. S6) include the NBD (region I); extended segments along the concave surfaces of CAP-D3 (region II) and CAP-G2 (region III); the C-terminal winged-helix domain (cWHD) contacting the distal surface of SMC4 (region IV-B); and an immediately N-terminal pair of α-helices (region IV-A) that appears to bridge the SMC4 head to CAP-D3.

Having established the protomer architecture, we next examined the inter-protomer contacts that organize the condensin II dimer. The dimer interface is formed by the SMC4 head domain and the CAP-H2 cWHD of one protomer, together with CAP-G2 HEAT repeats 11–13 and, to a lesser extent, the CAP-H2 BC2 segment of the other protomer (Fig. 1B; see Fig. S9 for HEAT-repeat numbering). The CAP-H2 BC2 loop projects into a groove on the apposing protomer, formed by the CAP-H2 pre-cWHD helix (region IV-A helix 2) and the subjacent SMC4 α_2_-helix, while the CAP-H2 BC1 segment tracks along the surface of CAP-G2 away from the dimer interface (see Figs. S7–S8 for the SMC helix numbering used here). The BC1 and BC2 segments of CAP-H2 are required for metaphase chromosome formation in human cells [29] and indeed for the association of condensin II with chromatin [23], but are buried in the dimer by the other protomer (for BC2) or by the surface of CAP-G2 HEAT repeat 10 (for BC1). Notably, the dimer interface is adjacent to the reported binding site for MCPH1 and M18BP1 [25], suggesting a possible role for these factors in regulating the oligomeric state of condensin II.

To determine whether the dimer we observed by cryo-EM is also present in solution, we measured the molecular-mass distribution of condensin II by mass photometry. Under the monovalent salt conditions which prevailed during purification (300 mM KCl), condensin II was exclusively monomeric (Fig. 1C, blue curve). Reducing the monovalent salt concentration to the physiological range (150 mM KCl) resulted in the appearance of an additional species with approximately twice the monomer mass (Fig. 1C, orange curve). Measurements over a broader range of salt concentrations showed that the dimer fraction increased as the salt concentration was lowered, and it approached a plateau at low salt (Fig. S10). Together, these data indicate that condensin II undergoes reversible, salt-dependent dimerization, with appreciable population of the dimeric state at physiological ionic strength.

The aforementioned burial of the essential CAP-H2 BC1 and BC2 segments at the dimer interface suggested that dimerization could restrain condensin II activity. This possibility was reinforced by another feature of the apoenzyme dimer: the CAP-H2 NBD, which corresponds to the kleisin domain that binds SMC2 to form the neck gate in structures of yeast condensin [11, 12], is not located at the SMC2 neck but is instead displaced from that site and sandwiched between the body of CAP-G2 and the C-terminal tail of CAP-D3 (Fig. 2). In this configuration, the convex face of CAP-G2 HEAT repeat 8 contacts the CAP-D3 HEAT docker, while the concave face of CAP-G2 HEAT repeats 6–7, together with the CAP-D3 C-terminal tail, sequesters the CAP-H2 NBD. This arrangement provides a structural basis for the proposed autoinhibitory role of the C-terminal tail of CAP-D3 [23, 24]. Previous structures of yeast condensin resolved the neck gate in closed states or inferred opening from loss of ordered density for the kleisin NBD in nucleotide-bound complexes with engaged SMC heads [11, 12]; by contrast, our structure directly resolves the displaced CAP-H2 NBD and reveals how CAP-G2 and CAP-D3 cooperate to stabilize an open-gate conformation.

**Figure 3:**
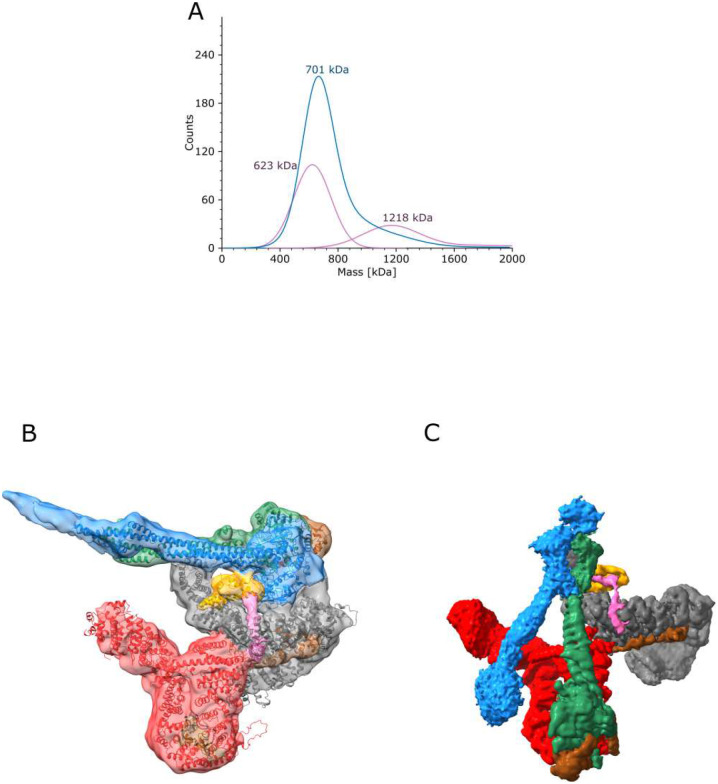
Nucleotide binding stabilizes the monomeric form of human condensin II. **(A)** Condensin II mass distributions in 87.5 mM KCl buffer with 0.025 mM ADP·BeF_3_ (purple) or 5 mM ADP·BeF_3_ (blue). Modal masses are indicated. **(B,C)** LocScale-sharpened cryo-EM maps of ADP·BeF_3_-bound monomeric condensin II **(B)** and one protomer from the apo dimer **(C)**, aligned on the HAWK module. In **(B)**, density is rendered transparently with the fitted model superposed in cartoon representation. The nucleotide-bound state exhibits a large-scale movement of the SMC module relative to the HAWK module. Subunit colors are the same in both panels: SMC2, blue; SMC4, green; CAP-H2, brown (with NBD in yellow); CAP-D3, red (with C-terminal tail in magenta); CAP-G2, gray.

Nucleotide binding is known to induce engagement of the condensin SMC heads [11, 12]. Because the SMC4 head forms part of the apo dimer interface, we reasoned that nucleotide-driven head engagement might perturb dimerization. We therefore asked how the addition of ADP·BeF_3_ would affect the oligomeric distribution of condensin II. Analysis by mass photometry showed that condensin II remained a mixture of monomeric and dimeric species in the presence of micromolar concentrations of ADP·BeF_3_ (Fig. 3A, purple curve), whereas millimolar concentrations of ADP·BeF_3_ shifted the distribution to the monomeric state (Fig. 3A, blue curve). Consistent with apparent nucleotide-dependent stabilization of the monomeric state, single-particle cryo-EM analysis of condensin II prepared with 5mm ADP·BeF_3_ yielded a monomeric reconstruction at 7.2 Å resolution (Fig. 3B and Fig. S11). As expected [11, 12], the SMC heads are engaged, although the local resolution does not permit assignment of the bound nucleotides. To visualize the nucleotide-dependent rearrangement, we compared the ADP·BeF_3_-bound monomer (Fig. 3B) with one protomer from the apo dimer after aligning the HAWK module (Fig. 3C). Relative to the dimer structure, CAP-D3 and CAP-G2 retain the same mutual arrangement, whereas the SMC module rotates with respect to them by approximately 90 degrees. This rotation shifts the SMC heads away from CAP-D3 and toward CAP-G2, disrupting the apo-state interaction between the CAP-D3 KG loop and the SMC4 W loop and bringing the SMC2 neck into close proximity with the CAP-H2 NBD. The neck gate, however, remains open: the CAP-D3 C-terminal tail continues to pin the CAP-H2 NBD against HEAT repeats 6–7 of CAP-G2, leaving the NBD sequestered in the same site observed in the apoenzyme. Thus, nucleotide binding stabilizes a monomeric state with engaged SMC heads, but does not release the CAP-H2 NBD from its autoinhibitory binding site.

The outcome of this rearrangement resembles the “flip-flop” mechanism described for yeast condensin, in which nucleotide binding changes the HEAT-repeat subunit associated with the SMC heads [11]. However, the apparent mechanism differs: in human condensin II, the HAWK module remains comparatively fixed, while the SMC module rotates with respect to it. In this respect, nucleotide-bound human condensin II also differs from nucleotide-bound yeast condensin, in which the neck gate is open but the kleisin NBD is not resolved, presumably because of flexibility [11, 12].

**Figure 4:**
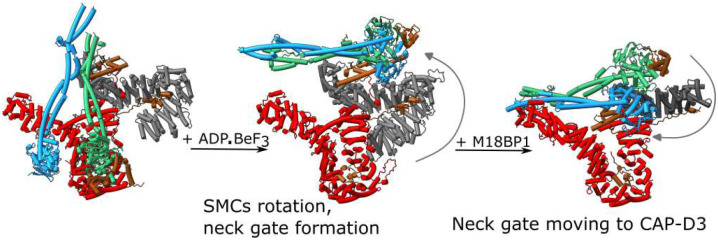
Proposed mechanism of activation of condensin II. Representation of major steps in condensin II activation, based on three cryo-EM structures. ADP·BeF_3_ binding rotates the SMC module relative to the HAWK module, bringing the SMC2 neck and CAP-H2 NBD into juxtaposition. This reconstitutes the neck gate, but the gate remains open due to continued blockade of the CAP-H2 NBD by the C-terminal tail of CAP-D3. Subsequent M18BP1 association induces a change in the HEAT-repeat subunit of the head module, resulting in a poised, pre-clamping intermediate (from left to right: apo condensin II [PDB 12FY], condensin II–ADP·BeF_3_ [PDB 12GD], M18BP1-bound condensin II–AMP-PNP [PDB 9F5W] [25]).

Subsequent addition of M18BP1 drives a further rearrangement of the SMC module relative to the HAWK module, placing CAP-G2 near the SMC4 W loop and repositioning CAP-D3 behind SMC2 [25] (Fig. 4). In overall architecture, this state resembles the DNA-clamped conformation of yeast condensin [12] and may therefore represent a poised, pre-clamping intermediate. Although the CAP-D3 C terminus is not resolved in the M18BP1-bound structure [25], leaving it unclear whether the CAP-H2 NBD remains pinned, the position of the CAP-H2 NBD relative to the SMC2 neck is essentially unchanged from the nucleotide-bound monomer.

In conclusion, our analysis reveals the structural basis for the condensin II autoinhibitory mechanism identified by Yoshida *et al*. [23, 24]: the CAP-D3 C-terminal tail pins the CAP-H2 NBD against the body of CAP-G2, maintaining an open neck gate. Monomerization, favored by nucleotide binding, liberates the CAP-H2 BC2 segment for presumptive DNA binding and causes a large-scale rotation of SMCs relative to the HEAT-repeat subunits, bringing the CAP-H2 NBD into proximity with the SMC2 neck, although the neck gate remains open (Fig. 4, first arrow). Finally, the addition of M18BP1, which binds to the convex surface of HEAT repeats 11–12 of CAP-G2, coincides with further movement of the SMCs relative to the fixed HAWK module, bringing CAP-D3 behind the SMCs in a manner suggestive of a poised, pre-clamping intermediate (Fig. 4, second arrow; Mov. S1).

An important open question is the identity of factors that stabilize the condensing II dimer, inasmuch as the intranuclear free concentration of ATP (approximately 4 mM [30]) would be expected to strongly bias the *in vivo* oligomeric-state distribution in favor of the monomer. In this regard, MCPH1 and M18BP1 are of particular interest, as both bind CAP-G2 near the observed dimer interface.

Although dimeric SMC-kleisin assemblies have previously been proposed in cohesin handcuff models of sister-chromatid cohesion [31] and in studies of regulated cohesin self-association [32], the condensin II dimer described here instead bears the hallmarks of an autoinhibited state, including occlusion of CAP-H2 elements required for chromosome association and sequestration of the CAP-H2 NBD away from the SMC2 neck. It is also tempting to speculate that the condensin II dimer we have observed could represent a stable, non-extrusive state, perhaps anchoring the bases of chromatin loops in condensed chromosomes.

## Materials and Methods

### Protein expression and purification

Human condensin II was cloned and purified as previously described [33]. Briefly, condensin II subunits were assembled into biGBac vectors, transposed into the baculoviral genome in DH10Bac cells, and then transfected into Sf9 insect cells. Virus was amplified in Sf9 and then used to infect High Five cells at a density of 10^6^ cells/mL. Infected High Five cells were resuspended in lysis buffer (50 mM HEPES [pH 8], 300 mM KCl, 5 mM MgCl_2_, 10% v/v glycerol) supplemented with 10 mM β-mercaptoethanol, protease inhibitors, and 25 U/mL of Turbonuclease, and lysed by sonication. The cleared lysate was loaded onto a Strep-Tactin XT column, which was subsequently washed with lysis buffer and eluted using the same buffer supplemented with 50 mM biotin. The eluate was diluted two-fold with buffer A (50 mM HEPES [pH 8], 5 mM MgCl_2_, 5% v/v glycerol, 1 mM DTT), loaded onto Heparin HP resin, washed with buffer A containing 250 mM NaCl, and eluted with buffer A containing 500 mM NaCl. Finally, the complex was loaded onto a Superose 6 Increase 10/300 column equilibrated with lysis buffer. Purified condensin II complexes were analyzed by SDS-PAGE (Fig. S1) and mass photometry (Fig. 1C).

### Nucleotide binding

To prepare the nucleotide-bound complex, samples of purified condensin II were supplemented with 5 mM ADP, 1 mM BeSO_4_, and 10 mM NaF [12], and allowed to incubate at room temperature for 30 min before use.

### Mass photometry

Mass photometry was performed on a TwoMP device (Refeyn). Protein was diluted with lysis buffer before measurement and deposited onto uncoated glass slides (Refeyn MGUC, MP-CON-21022) overlaid with 6×3.0-mm silicone gaskets (Refeyn RD501078). The data (movies of 1 min duration) were acquired with AcquireMP (Refeyn) and analyzed with DiscoverMP (Refeyn) software packages. MassFerence P1 Calibrant (Refeyn MP-CON-41033) was used to construct a calibration curve.

### Cross-linking

Purified condensin II was exchanged into an aqueous buffer consisting of 50 mM HEPES (pH 8), 100 mM KCl, 1 mM DTT, and 5 mM MgCl_2_, and then concentrated to 1 μM using a centrifugal filter device. For nucleotide-containing samples, 5 mM ADP, 1 mM BeSO_4_, and 10 mM NaF were then added, followed by incubation for 30 min at room temperature. BS3 (bis(sulfosuccinimidyl)suberate) was added to a final concentration of 0.5 mM, and the sample was incubated for 30 min at room temperature. Unreacted cross-linker was quenched by addition of a 100-fold molar excess of Tris (pH 8).

### Vitrification

Quantifoil R2/1 200-mesh copper grids were glow discharged for 45 s at 0.4 mbar and 15 mA in a PELCO easiGlow glow discharge cleaning system (Ted Pella, Inc.). Grids were loaded into the humidified chamber of a Vitrobot Mark IV (Thermo Fisher Scientific) maintained at 4°C and 100% relative humidity. A 3 μL volume of sample solution was directly applied onto each grid followed by blotting for 2 seconds and then plunging into liquid ethane. The frozen grids were stored in liquid nitrogen for further screening and data collection.

### Cryo-EM imaging and data processing

Data for apo-condensin II were collected on a 300-kV Titan Krios electron microscope (Thermo Fisher Scientific) equipped with a K3 direct electron detector camera (Gatan Inc.), operated in counting mode, and a BioQuantum energy filter (Gatan Inc.) (20 eV in slit width). Acquisition settings included an exposure time of 5 seconds, a total dose of 51 e/Å^2^, 50 total frames for each movie-mode stack, a magnification of 64,000×, a pixel size of 1.4 Å, and a defocus range of −1.0 to −2.4 μm. A representative micrograph is shown in Fig. S2A. The monomer data were collected on a 200-kV Glacios G2 electron microscope (Thermo Fisher Scientific) equipped with a Falcon 4i camera and a Selectris energy filter (15 eV in slit width). Acquisition settings included an exposure time of 12.58 seconds, a total dose of 50 e/Å^2^, 40 total frames for each movie-mode stack, a magnification of 100,000×, a pixel size of 1.18 Å, and a defocus range of −1.2 to −2.4 μm. A representative micrograph is shown in Fig. S11A. All datasets were processed using cryoSPARC [34] and RELION [35]. MotionCor2 [36] was used for motion correction after importing movie-mode images into RELION, and CTFFIND4 [37] was used for estimation of the contrast transfer function (CTF). Pyem [38] was used to convert the particles processed in cryoSPARC for further processing in RELION. After CTF estimation in RELION, micrographs with poor CTF fits were manually excluded using RELION’s Subset Selection job. The dimer particles were initially selected from a subset of micrographs using RELION’s Laplacian-of-Gaussian blob-picking feature and then subjected to 2D classification in cryoSPARC. The monomer particles were initially selected from a subset of micrographs using the dimer’s 2D class averages as templates. The final sets of particles were selected with Topaz [39], using a model trained on a curated set of good particles. The particles were initially extracted with 4× (or 2×) binning in RELION, and then imported into cryoSPARC for 2D classification and Heterogeneous Refinement to eliminate “junk” particles and low-quality classes. After cryoSPARC Heterogeneous Refinement, particles from selected classes were converted to RELION format using csparc2star.py [38], and then re-extracted without binning. Gold-standard refinement of the final maps was performed using RELION’s 3D auto-refine or cryoSPARC’s Non-Uniform Refinement or Local Refinement. Flow charts depicting data-processing steps are included in Fig. S2B (for apo dimer), Fig. S4A (for coiled coil and hinge domains), and Fig. S11B (for nucleotide-bound monomer). Gold-standard Fourier shell correlation (FSC) curves, viewing direction distributions, and local resolution estimates are included in Fig. S2C–E (for apo dimer), Fig. S4B–G (for coiled coil and hinge domains), and Fig. S11C–E (for nucleotide-bound monomer). Data collection and processing statistics are included in Table S1 (for apo dimer) and Table S2 (for nucleotide-bound monomer). For figure preparation, local map sharpening was performed with LocScale 2.0 in hybrid mode using the unsharpened half-maps and the final refined atomic model as input [40–42]. Refmac refinement was disabled during LocScale processing.

### Model building

The sequences of the human condensin II subunits were used to predict AlphaFold 3 multimeric models using the AlphaFold server (https://alphafoldserver.com/) [43]. The models found to be most useful and therefore used for subsequent model building were CAP-D3/CAP-G2/CAP-H2, CAP-H2/SMC4, SMC2/SMC4, and SMC2. Models were initially fit into the maps using ChimeraX [44], and the best-fitting parts of the various models were combined into a single model. The model’s initial fit was then improved using ISOLDE [45]. Local anisotropic map sharpening was performed in Phenix, and the resulting maps were used for subsequent refinement and model building [46]. Subsequent rounds of manual improvement in Coot, ISOLDE, and Phenix real-space refinement were performed.

## Supplementary Material

1

## Data Availability

The cryo-EM maps generated in this study have been deposited in the Electron Microscopy Data Bank under accession codes EMD-76412, EMD-76413, EMD-76414, and EMD-76416. Atomic coordinates have been deposited in the Protein Data Bank under accession codes 12FY, 12FZ, 12GA, and 12GD. These entries correspond, respectively, to the human condensin II apo form dimer, the coiled-coil focused reconstruction, the elbow/hinge focused reconstruction, and condensin II-ADP·BeF_3_.

## Code availability

No custom code was generated for this study.
