# Supplementary material for: Structural basis of condensin II activation
Source: Res Sq. 2026 Jun 5:rs.3.rs-9335492. Preprint. [Version 1] doi: 10.21203/rs.3.rs-9335492/v1 (PMC13252511; doi:10.21203/rs.3.rs-9335492/v1)
Supplement: 1 [file NIHPPRS9335492V1-supplement-1.pdf]

Supplementary Information

Contents

Figures S1–S11
Movie S1
Tables S1–S2

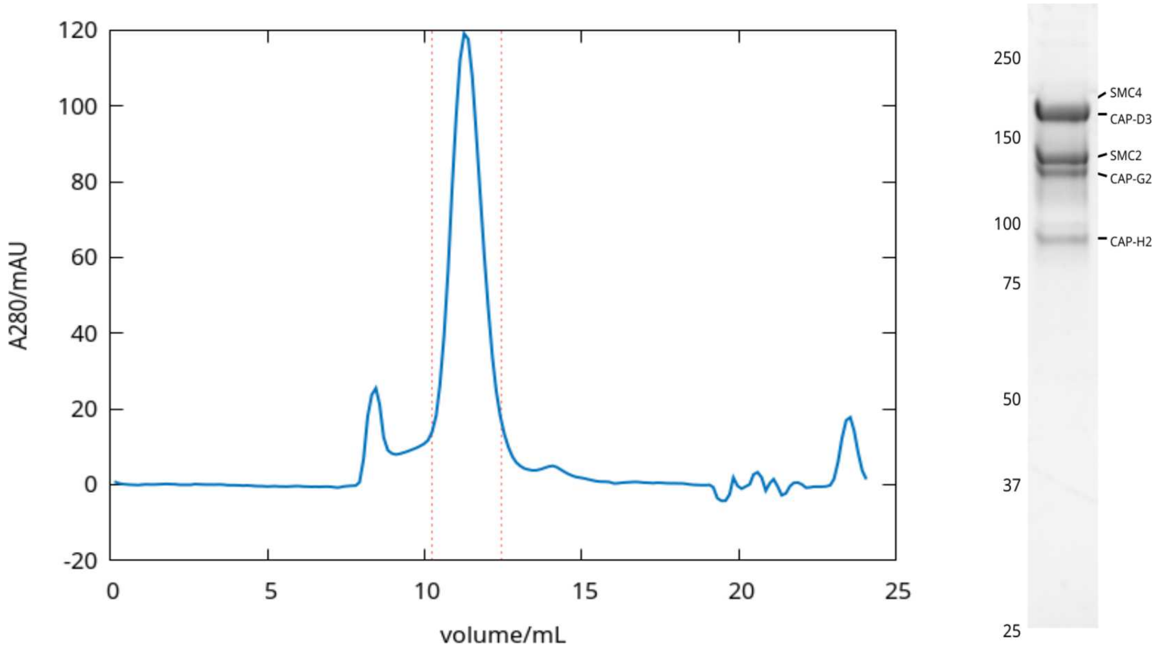

Figure S1: Purification of human condensin II. (Left) Chromatogram of human condensin II purified using a Superose 6 Increase 10/300 GL column. (Right) Denaturing gel of the pooled fractions of the main peak, with subunit identities and molecular-weight markers indicated.

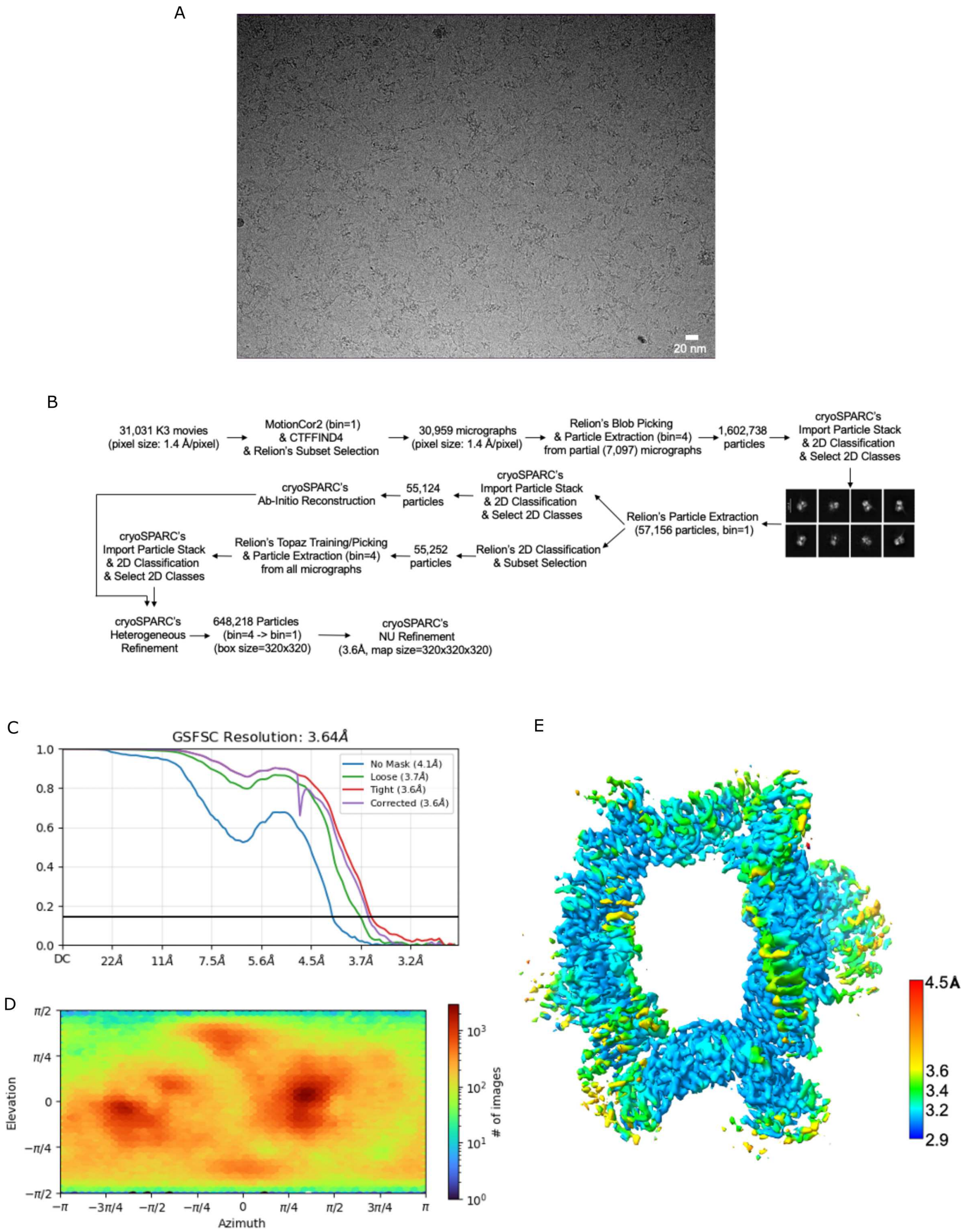
A
20 nm
B
31,031 K3 movies (pixel size: 1.4 Å/pixel)
MotionCor2 (bin=1) & CTFFIND4 & Relion's Subset Selection
30,959 micrographs (pixel size: 1.4 Å/pixel)
Relion's Blob Picking & Particle Extraction (bin=4) from partial (7,097) micrographs
1,602,738 particles
cryoSPARC's Import Particle Stack & 2D Classification & Select 2D Classes
Relion's Particle Extraction (57,156 particles, bin=1)
cryoSPARC's Import Particle Stack & 2D Classification & Select 2D Classes
55,124 particles
cryoSPARC's Ab-Initio Reconstruction
Relion's 2D Classification & Subset Selection
55,252 particles
Relion's Topaz Training/Picking & Particle Extraction (bin=4) from all micrographs
cryoSPARC's Import Particle Stack & 2D Classification & Select 2D Classes
cryoSPARC's Heterogeneous Refinement
648,218 Particles (bin=4 -> bin=1) (box size=320x320)
cryoSPARC's NU Refinement (3.6Å, map size=320x320x320)
C
GSFSC Resolution: 3.64Å
No Mask (4.1Å)
Loose (3.7Å)
Tight (3.6Å)
Corrected (3.6Å)
1.0
0.8
0.6
0.4
0.2
0.0
DC
22Å
11Å
7.5Å
5.6Å
4.5Å
3.7Å
3.2Å
D
Elevation
π/2
π/4
0
−π/4
−π/2
−π
−3π/4
−π/2
−π/4
0
π/4
π/2
3π/4
π
Azimuth
# of images
10^3
10^2
10^1
10^0
E
4.5Å
3.6
3.4
3.2
2.9

Figure S2: Cryo-EM data processing for apo human condensin II. (A) Representative micrograph of apo condensin II collected on a Titan Krios equipped with a K3 camera. The micrograph was motion-corrected and dose-weighted and had an estimated defocus of $-2.3\,\mu m$. Scale bar, 20 nm. (B) Data-processing workflow for the apo condensin II reconstruction. A subset of micrographs was used for blob picking and 2D classification, and selected 2D classes were used for *ab initio* reconstruction in cryoSPARC and for Topaz training and particle picking in RELION. A total of 648,218 particles extracted with a 320×320-pixel box were used to reconstruct the dimer map. (C) Gold-standard Fourier shell correlation (FSC) curves for the final reconstruction of the apo condensin II dimer. (D) Distribution of viewing directions. (E) Cryo-EM map colored according to the estimated local resolution.

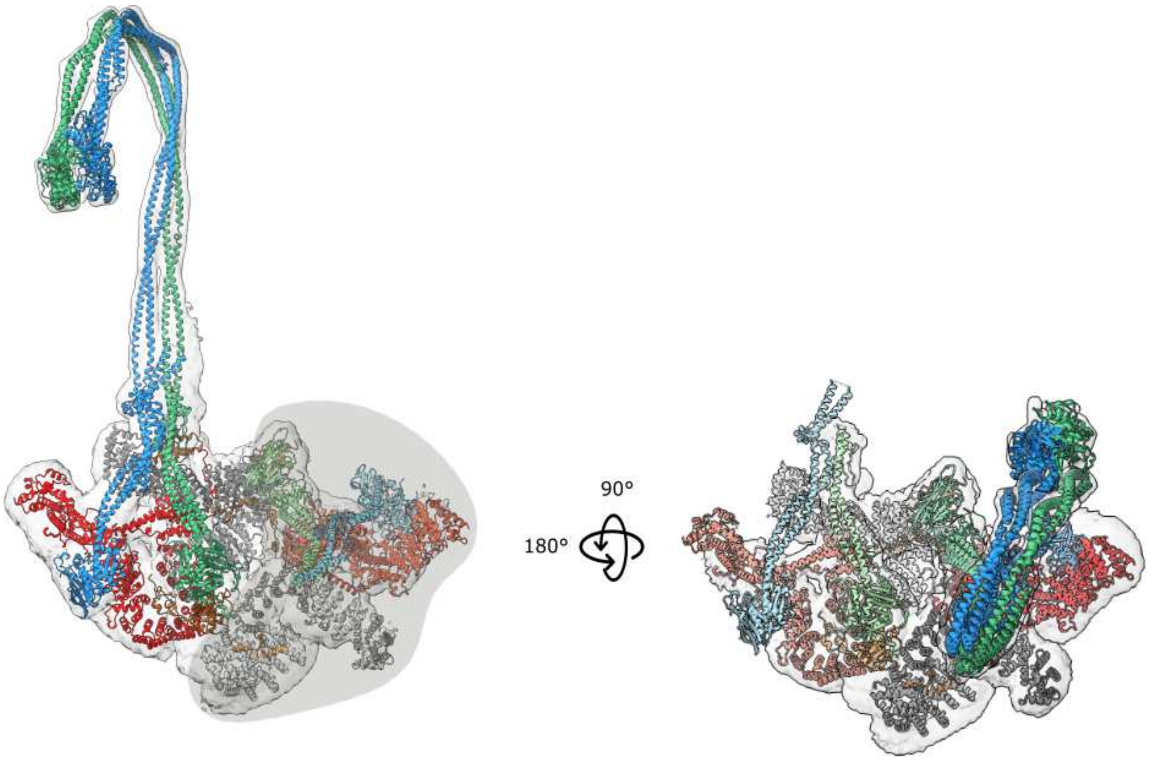

Figure S3: Composite map of human condensin II. Composite map, prepared in ChimeraX, of PDB 12FY, PDB 12FZ, and PDB 12GA showing condensin II in its apo form as a dimer with one coiled-coil/hinge region visible. One protomer is highlighted in gray, while the other is unshaded. The dimer exhibits pseudo-C2 symmetry. Subunits are colored as follows: SMC2, blue; SMC4, green; CAP-H2, brown; CAP-D3, red; CAP-G2, gray.

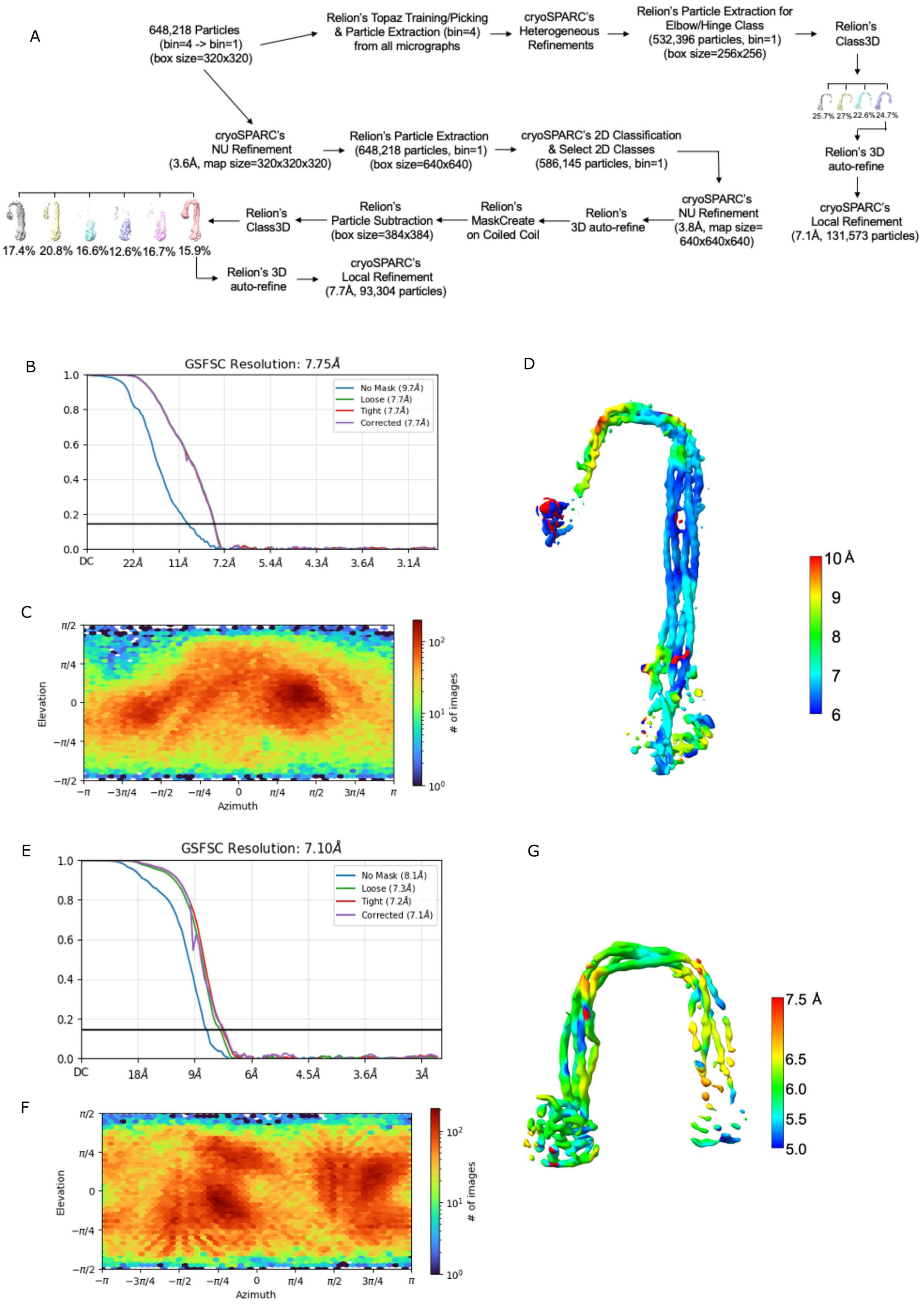
A
648,218 Particles (bin=4 -> bin=1) (box size=320x320)
Relion's Topaz Training/Picking & Particle Extraction (bin=4) from all micrographs
cryoSPARC's Heterogeneous Refinements
Relion's Particle Extraction for Elbow/Hinge Class (532,396 particles, bin=1) (box size=256x256)
Relion's Class3D
25.7% 27% 22.6% 24.7%
Relion's 3D auto-refine
cryoSPARC's Local Refinement (7.1Å, 131,573 particles)
cryoSPARC's NU Refinement (3.6Å, map size=320x320x320)
Relion's Particle Extraction (648,218 particles, bin=1) (box size=640x640)
cryoSPARC's 2D Classification & Select 2D Classes (586,145 particles, bin=1)
cryoSPARC's NU Refinement (3.8Å, map size= 640x640x640)
Relion's 3D auto-refine
Relion's MaskCreate on Coiled Coil
Relion's Particle Subtraction (box size=384x384)
Relion's Class3D
17.4% 20.8% 16.6% 12.6% 16.7% 15.9%
Relion's 3D auto-refine
cryoSPARC's Local Refinement (7.7Å, 93,304 particles)
B
GSFSC Resolution: 7.75Å
No Mask (9.7Å)
Loose (7.7Å)
Tight (7.7Å)
Corrected (7.7Å)
DC 22Å 11Å 7.2Å 5.4Å 4.3Å 3.6Å 3.1Å
C
Elevation
Azimuth
# of images
D
10 Å
9
8
7
6
E
GSFSC Resolution: 7.10Å
No Mask (8.1Å)
Loose (7.3Å)
Tight (7.2Å)
Corrected (7.1Å)
DC 18Å 9Å 6Å 4.5Å 3.6Å 3Å
F
Elevation
Azimuth
# of images
G
7.5 Å
6.5
6.0
5.5
5.0

Figure S4: Focused cryo-EM refinement and validation of the apo condensin II coiled-coil and hinge regions. (A) Workflow for focused classification and refinement of the coiled-coil and hinge regions. Particles from the apo dimer reconstruction were re-extracted with a 640×640-pixel box to include the coiled-coil and hinge regions, and a corresponding 3D reconstruction was generated. A mask around the coiled coils was used for focused 3D classification in RELION, and selected particles were refined in cryoSPARC. The elbow/hinge class was selected during cryoSPARC heterogeneous refinement and refined separately. (B) Gold-standard FSC curves for the coiled-coil reconstruction. (C) Distribution of viewing directions for the coiled-coil reconstruction. (D) Local-resolution estimate for the coiled-coil reconstruction. (E) Gold-standard FSC curves for the hinge reconstruction. (F) Distribution of viewing directions for the hinge reconstruction. (G) Local-resolution estimate for the hinge reconstruction.

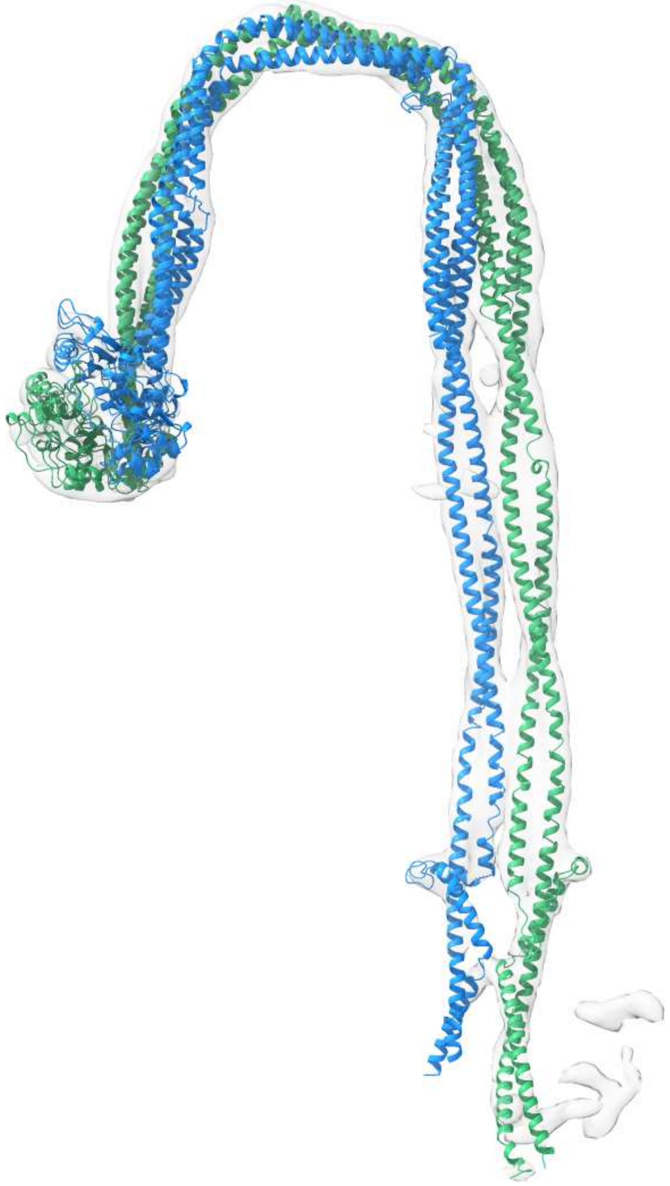

Figure S5: Human condensin II coiled-coil and hinge domains. Overlay of PDB 12FZ and PDB 12GA showing the SMC coiled-coil and hinge domains reconstructed by focused refinement of the apo condensin II dimer. SMC2 and SMC4, shown in cartoon representation, are colored blue and green, respectively.

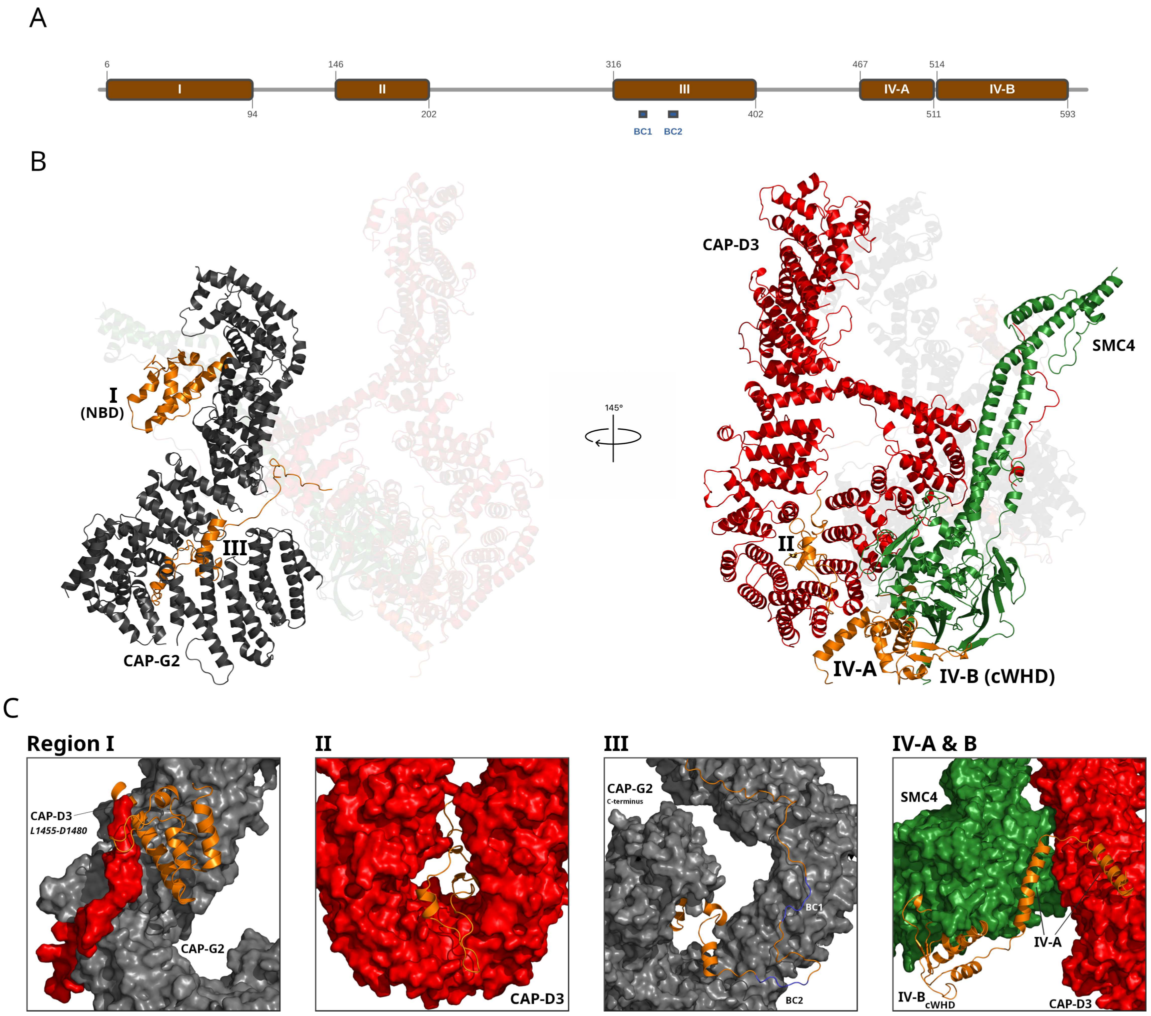
A
6
146
316
467
514
I
II
III
IV-A
IV-B
94
202
402
511
593
BC1
BC2
B
CAP-D3
SMC4
I
(NBD)
145°
III
II
CAP-G2
IV-A
IV-B (cWHD)
C
Region I
II
III
IV-A & B
CAP-D3
L1455-D1480
CAP-G2
CAP-D3
CAP-G2
C-terminus
BC1
BC2
SMC4
IV-A
IV-B
cWHD
CAP-D3

Figure S6: Domain organization and intraprotomeric contacts of CAP-H2 in the apo condensin II dimer. **(A)** Domain organization of human CAP-H2, divided into regions I–IV according to resolved contacts with CAP-G2, CAP-D3, and SMC4. The positions of the BC1 and BC2 segments within region III are denoted by blue boxes. **(B)** Regions I–IV highlighted in CAP-H2 from one protomer of the human condensin II dimer, shown in two orientations; SMC2 is omitted for clarity. **(C)** Magnified views of the four regions, with CAP-H2 shown in cartoon representation, and non-CAP-H2 subunits shown as molecular surfaces. Region I (residues 6–94) contains the SMC2 neck-binding domain and is sandwiched by the CAP-D3 C-terminal tail and CAP-G2. Region II (residues 146–202) contacts the concave surface of CAP-D3. Region III (residues 316–402) contains the CAP-G2-binding domain, which includes the BC1 and BC2 segments (shaded in blue). Region IV-A (residues 467–511) comprises a pair of CAP-H2 α-helices, with one helix contacting the SMC4 head and the other contacting CAP-D3, thereby spanning the intraprotomeric SMC4–CAP-D3 interface. Region IV-B (residues 514–593) corresponds to the CAP-H2 C-terminal winged-helix domain (cWHD), which contacts the distal surface of the SMC4 head domain.

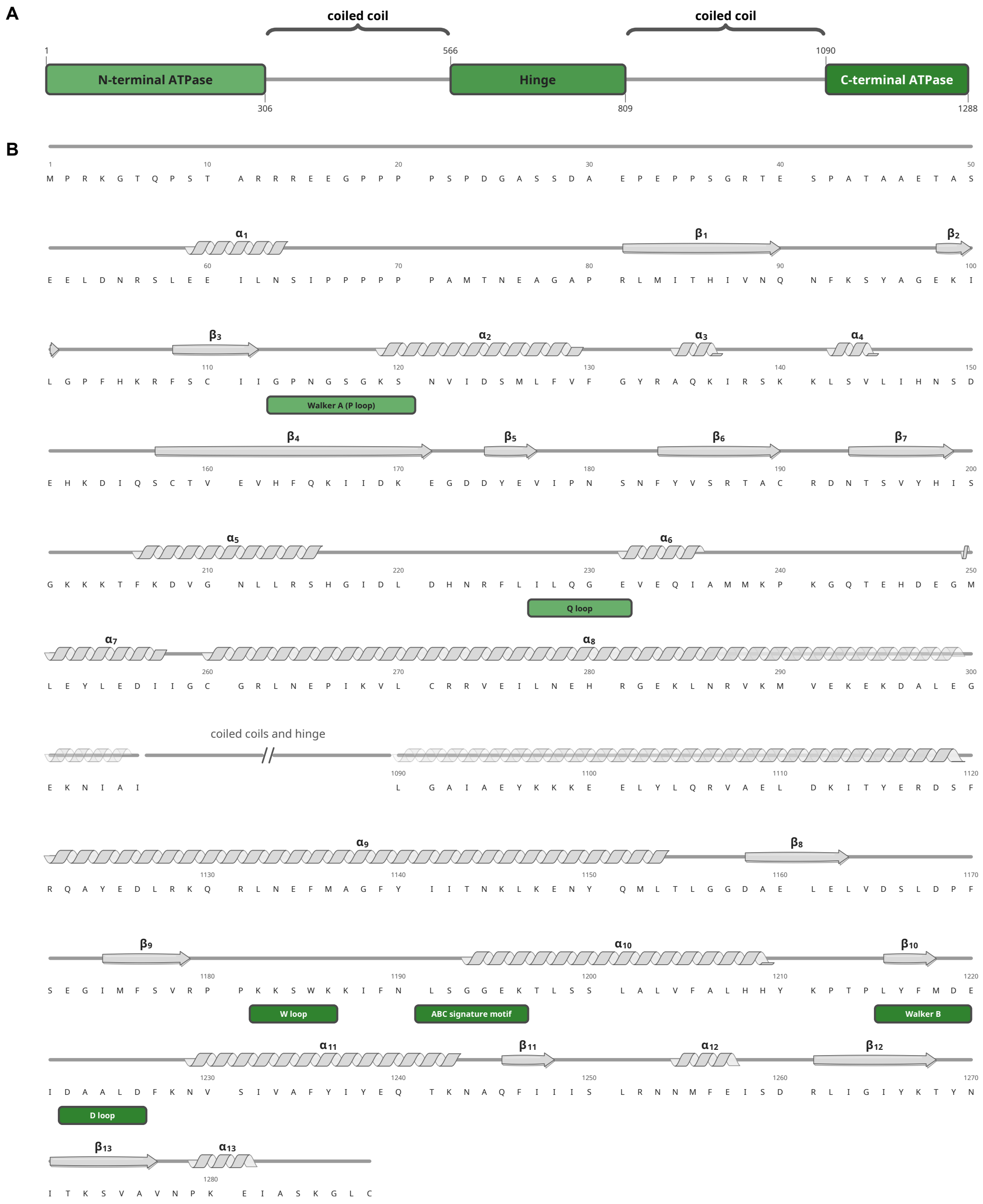
A
coiled coil
coiled coil
1
566
1090
N-terminal ATPase
Hinge
C-terminal ATPase
306
809
1288
B
Walker A (P loop)
Q loop
coiled coils and hinge
W loop
ABC signature motif
Walker B
D loop

Figure S7: Domain organization and ATPase-motif map of human SMC4. (A) Schematic domain organization of human SMC4, showing the N-terminal and C-terminal ATPase-head segments separated by the coiled-coil arms and hinge domain. **(B)** Sequence-resolved map of the SMC4 ATPase-head segments, with the intervening coiled-coil and hinge region collapsed. Secondary-structure elements are shown above the sequence, and conserved ATPase motifs, including Walker A (P loop), Q loop, W loop, ABC signature motif, Walker B, and D loop, are marked below the sequence.

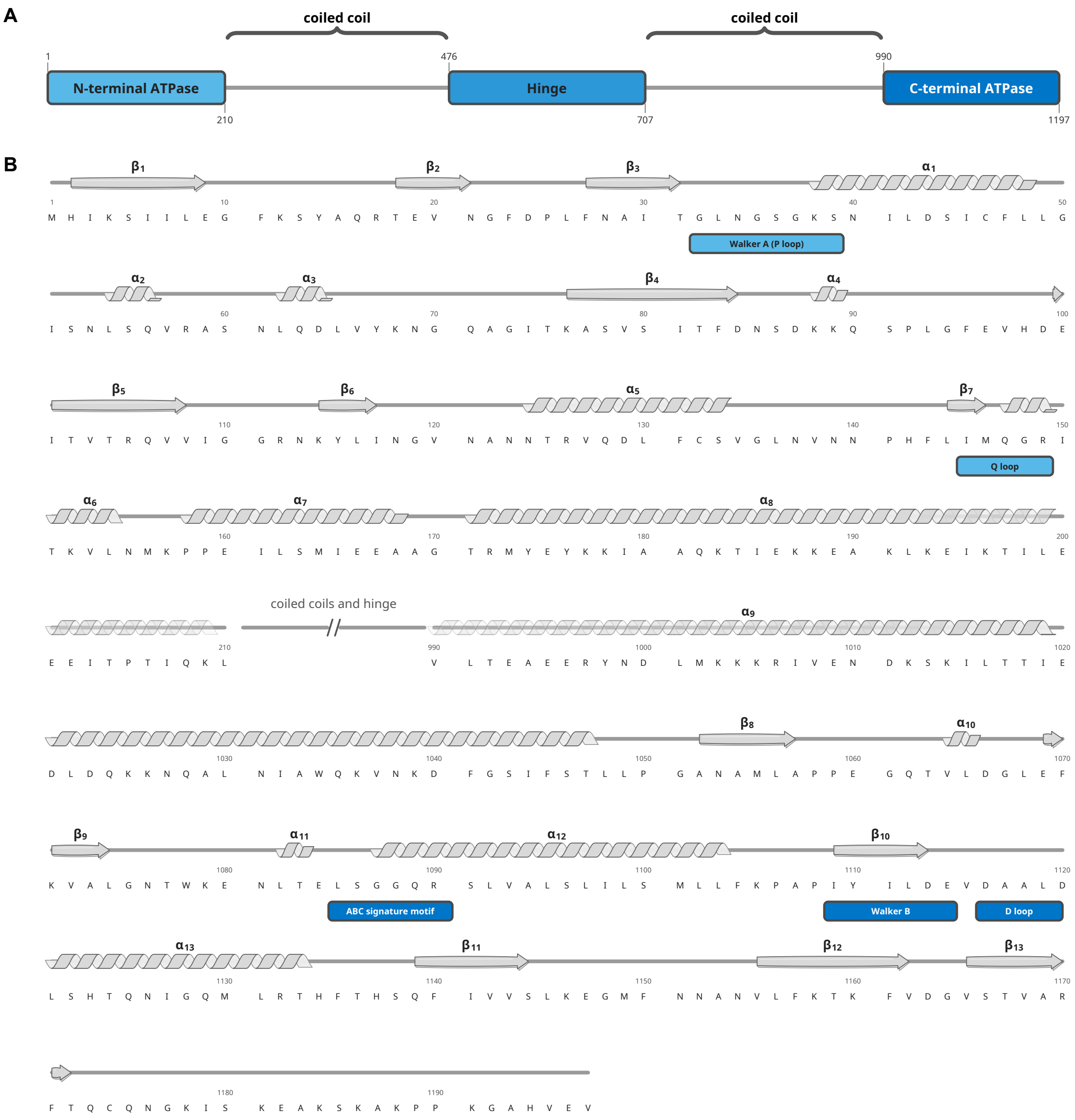
A
coiled coil
coiled coil
1
476
990
N-terminal ATPase
Hinge
C-terminal ATPase
210
707
1197
B
MHIKSIILEG FKSYAQRTEV NGFDPLFNAI TGLNGSGKSN ILDSICFLLG
Walker A (P loop)
ISNLSQVRAS NLQDLVYKNG QAGITKASVS ITFDNSDKKQ SPLGFEVHDE
ITVTRQVVIG GRNKYLINGV NANNTRVQDL FCSVGLNVNN PHFLIMQGRI
Q loop
TKVLNMKPPE ILSMIEEAAG TRMYEYKKIA AQKTIEKKEA KLKEIKTILE
coiled coils and hinge
EEITPTIQKL V LTEAEERYND LMKKKRIVEN DKSKILTTIE
DLDQKKNQAL NIAWQKVNKD FGSIFSTLLP GANAMLAPPE GQTVLDGLEF
KVALGNTWKE NLTELSGGQR SLVALSLILS MLLFKPAPIY ILDEVDAALD
ABC signature motif
Walker B
D loop
LSHTQNIGQM LRTHFTHSQF IVVSLKEGMF NNANVLFKTK FVDGVSTVAR
FTQCQNGKIS KEAKSKAKPP KGAHVEV

Figure S8: Domain organization and ATPase-motif map of human SMC2. **(A)** Schematic domain organization of human SMC2, showing the N-terminal and C-terminal ATPase-head segments separated by the coiled-coil arms and hinge domain. **(B)** Sequence-resolved map of the SMC2 ATPase-head segments, with the intervening coiled-coil and hinge region collapsed. Secondary-structure elements are shown above the sequence, and conserved ATPase motifs, including Walker A (P loop), Q loop, ABC signature motif, Walker B, and D loop, are marked below the sequence.

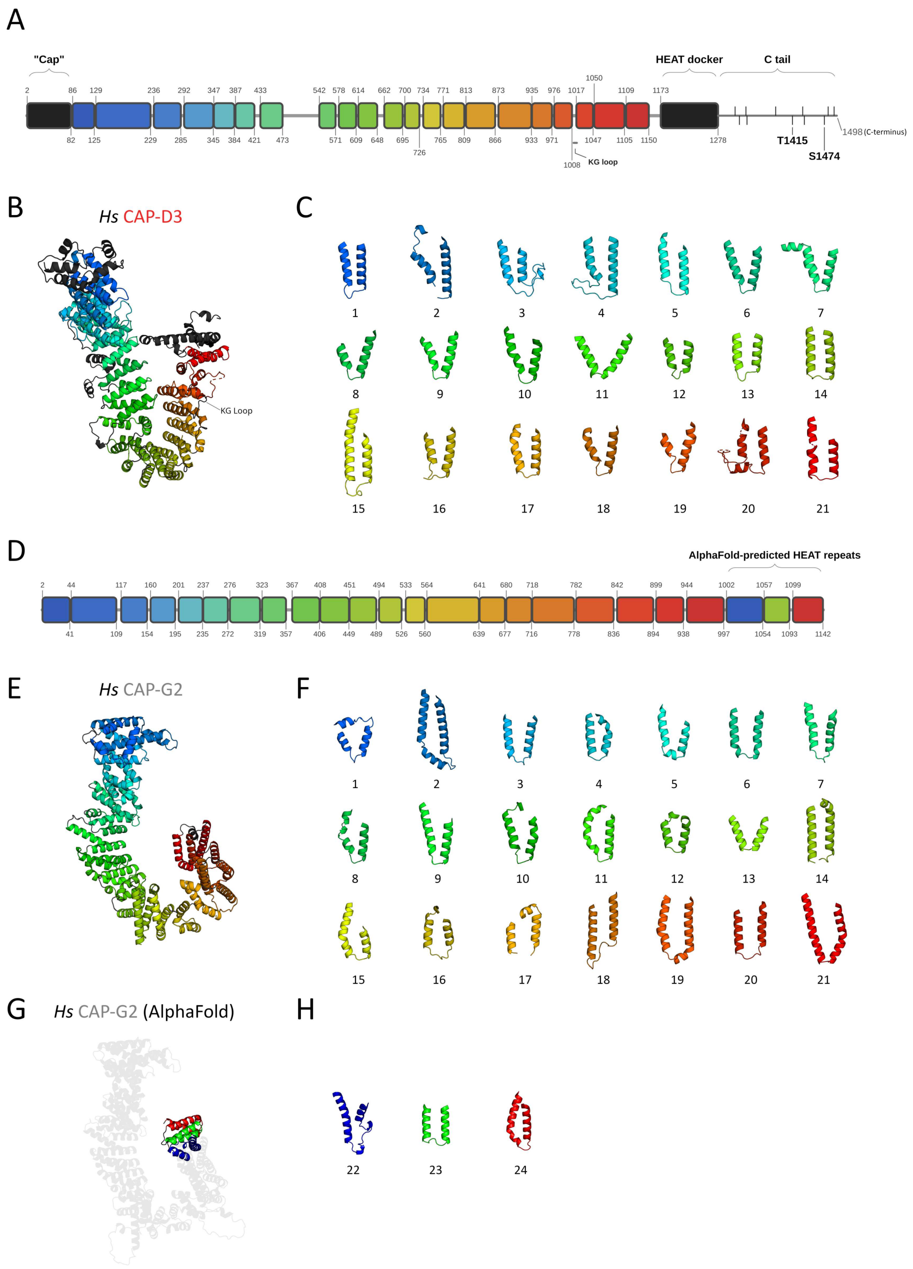
A
"Cap"
HEAT docker
C tail
2
86
129
236
292
347
387
433
542
578
614
662
700
734
771
813
873
935
976
1017
1050
1109
1173
82
125
229
285
345
384
421
473
571
609
648
695
726
765
809
866
933
971
1008
KG loop
1047
1105
1150
1278
T1415
S1474
1498 (C-terminus)
B
Hs CAP-D3
KG Loop
C
1
2
3
4
5
6
7
8
9
10
11
12
13
14
15
16
17
18
19
20
21
D
AlphaFold-predicted HEAT repeats
2
44
117
160
201
237
276
323
367
408
451
494
533
564
641
680
718
782
842
899
944
1002
1057
1099
41
109
154
195
235
272
319
357
406
449
489
526
560
639
677
716
778
836
894
938
997
1054
1093
1142
E
Hs CAP-G2
F
1
2
3
4
5
6
7
8
9
10
11
12
13
14
15
16
17
18
19
20
21
G
Hs CAP-G2 (AlphaFold)
H
22
23
24

Figure S9: Domain organization and HEAT-repeat architecture of the condensin II HAWK subunits CAP-D3 and CAP-G2. (A) Domain organization of human CAP-D3. HEAT repeats 1–21 are segmented and colored from the N terminus to the C terminus, with the N-terminal "cap" and C-terminal "HEAT docker" shown in dark gray. Tick marks in the C-terminal tail mark consensus CDK1 phosphorylation sites, with T1415 and S1474 specifically indicated. The position of the KG loop is indicated. **(B)** and **(C)** CAP-D3 shown as a full model and as isolated HEAT repeats, colored from blue at the N terminus to red at the C terminus. The position of the KG loop is indicated. **(D)** Domain organization of human CAP-G2. HEAT repeats 1–21 are resolved in the cryo-EM structure, whereas repeats 22–24 are assigned from a model predicted by AlphaFold 3. **(E)** and **(F)** The first 21 HEAT repeats of CAP-G2 shown in the same N- to C-terminal color gradient. **(G)** and **(H)** AlphaFold-predicted HEAT repeats 22–24 of CAP-G2, shown separately and colored blue, green, and red, respectively.

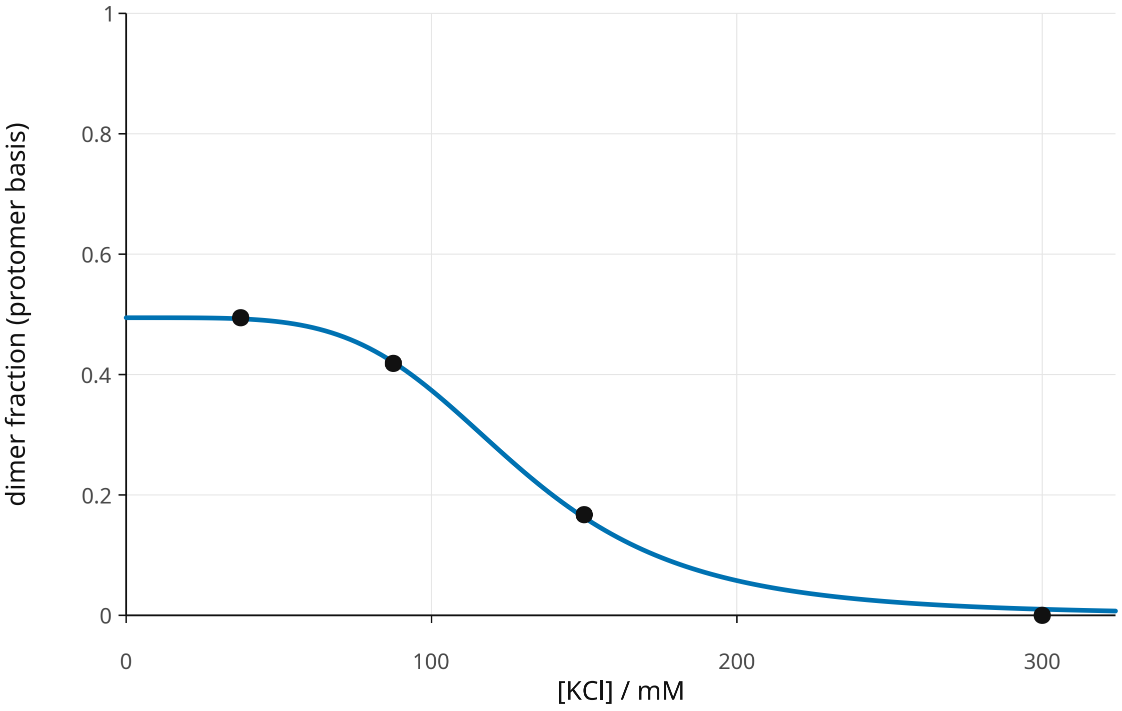

Figure S10: Salt-dependent dimerization of human condensin II measured by mass photometry. Fraction of protomers in dimers as a function of KCl concentration. Points represent mass-photometry measurements, and the superposed line shows a fit to the Hill model. The fit yielded a half-maximal KCl concentration of 130 mM and a Hill slope of 4.6.

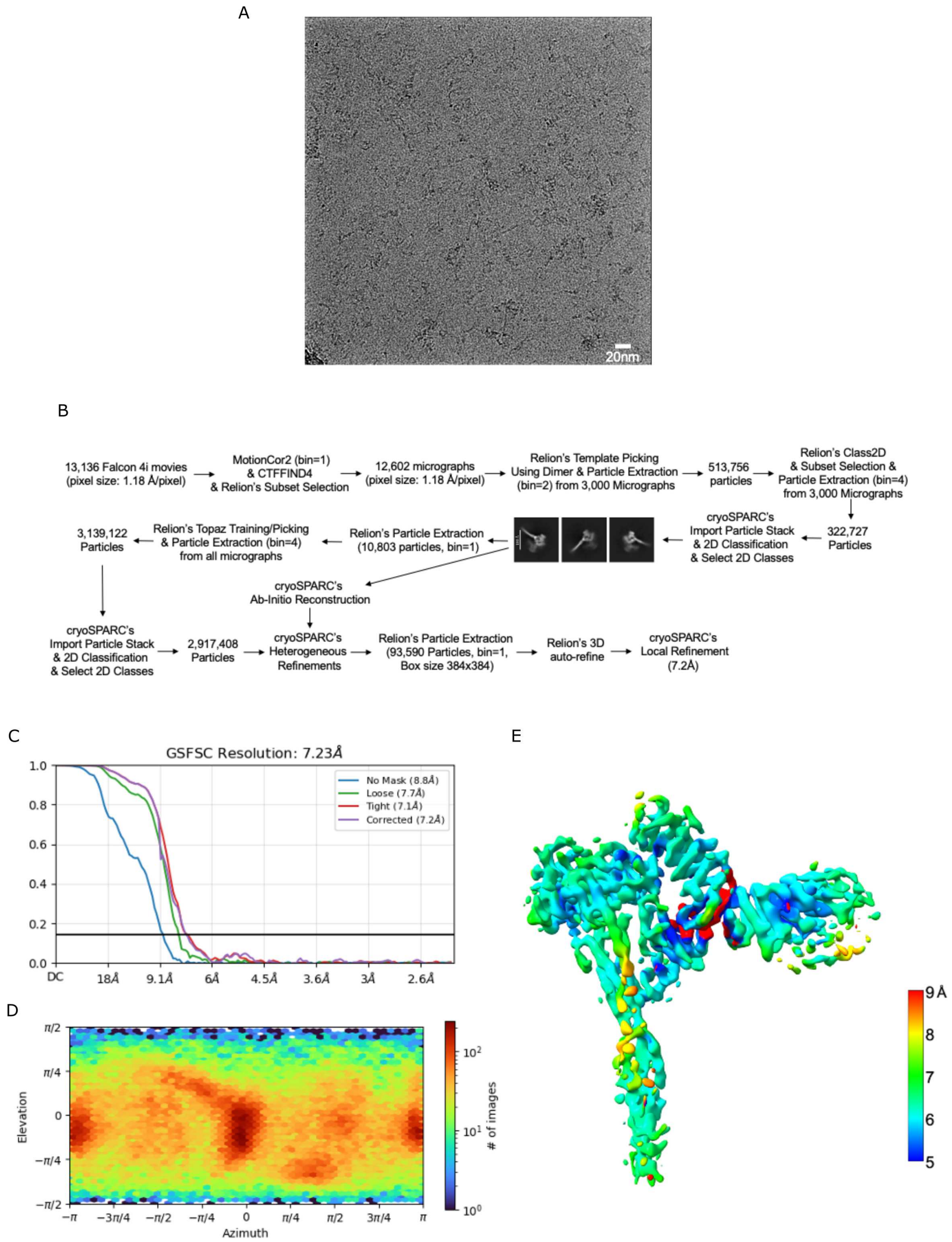
A
20nm
B
13,136 Falcon 4i movies (pixel size: 1.18 Å/pixel)
MotionCor2 (bin=1) & CTFFIND4 & Relion's Subset Selection
12,602 micrographs (pixel size: 1.18 Å/pixel)
Relion's Template Picking Using Dimer & Particle Extraction (bin=2) from 3,000 Micrographs
513,756 particles
Relion's Class2D & Subset Selection & Particle Extraction (bin=4) from 3,000 Micrographs
322,727 Particles
cryoSPARC's Import Particle Stack & 2D Classification & Select 2D Classes
Relion's Particle Extraction (10,803 particles, bin=1)
Relion's Topaz Training/Picking & Particle Extraction (bin=4) from all micrographs
3,139,122 Particles
cryoSPARC's Ab-Initio Reconstruction
cryoSPARC's Import Particle Stack & 2D Classification & Select 2D Classes
2,917,408 Particles
cryoSPARC's Heterogeneous Refinements
Relion's Particle Extraction (93,590 Particles, bin=1, Box size 384x384)
Relion's 3D auto-refine
cryoSPARC's Local Refinement (7.2Å)
C
GSFSC Resolution: 7.23Å
No Mask (8.8Å)
Loose (7.7Å)
Tight (7.1Å)
Corrected (7.2Å)
1.0
0.8
0.6
0.4
0.2
0.0
DC
18Å
9.1Å
6Å
4.5Å
3.6Å
3Å
2.6Å
D
Elevation
Azimuth
# of images
π/2
π/4
0
−π/4
−π/2
−π
−3π/4
−π/2
−π/4
0
π/4
π/2
3π/4
π
10^2
10^1
10^0
E
9 Å
8
7
6
5

Figure S11: Cryo-EM data processing for human condensin II-ADP·BeF$_3$. (A) Representative micrograph of condensin II-ADP·BeF$_3$ collected on a Glacios G2 equipped with a Falcon 4i camera. The micrograph was motion-corrected and dose-weighted and had an estimated defocus of $-2.3\,\mu$m. Scale bar, 20 nm. (B) Data-processing workflow for the condensin II-ADP·BeF$_3$ reconstruction. Apo-condensin II dimer 2D class averages were used as templates for particle picking, and selected 2D class averages were used for *ab initio* reconstruction in cryoSPARC and for Topaz training and particle picking in RELION. A total of 93,590 particles were used to reconstruct the monomer map, which was refined in cryoSPARC to 7.2 Å resolution. (C) Gold-standard Fourier shell correlation (FSC) curves for the final condensin II–ADP·BeF$_3$ reconstruction. (D) Distribution of viewing directions. (E) Cryo-EM map colored according to the estimated local resolution.

Movie S1: Proposed mechanism of activation of condensin II. Structural morph of the proposed activation pathway of human condensin II, proceeding through three conformational states: the apo condensin II dimer (PDB 12FY), condensin II–ADP·BeF_3 (PDB 12GD), and M18BP1-bound condensin II–AMP-PNP (PDB 9F5W) [25]. ADP·BeF_3 binding rotates the SMC module relative to the HAWK module, juxtaposing the SMC2 neck and CAP-H2 NBD without neck-gate closure, before M18BP1 association induces a change in the SMC-associated HAWK subunit, resulting in a poised, preclamping intermediate.

Table S1: Cryo-EM data collection, refinement, and validation statistics for the human condensin II apo form dimer and focused maps.

	Apo form dimer	Coiled Coil	Elbow/Hinge
**Data collection and processing**			
Microscope	Titan Krios		
Magnification	64,000		
Voltage (kV)	300		
Camera	Gatan K3		
Energy filter name	GIF Bioquantum		
Energy filter slit width	20 eV		
Automation software	SerialEM		
Electron exposure ($e^-/Å^2$)	51		
Defocus range (μm)	−1.0 to −2.4		
Pixel size (Å)	1.4		
Map sharpening B factor ($Å^2$)	−163.9	−691.8	−824.4
Map resolution (Å)	3.6	7.7	7.1
FSC threshold	0.143	0.143	0.143
EMDB entry	76412	76413	76414
Initial particle number	8,062,952	648,218	2,949,903
Final particle number	648,218	93,304	131,573
**Model Refinement**			
Model resolution (Å)	masked: 4.0 (3.6); unmasked: 4.2 (3.7)	masked: 9.3 (7.7); unmasked: 10.8 (8.1)	masked: 8.2 (7.2); unmasked: 8.4 (7.3)
FSC threshold	0.5 (0.143)	0.5 (0.143)	0.5 (0.143)
PDB entry	12FY	12FZ	12GA
**Model composition**			
Non-hydrogen atoms	58,171	13,680	8,058
Protein residues	7,228	1,695	1,011
Nucleotides	0	0	0
Ligands	0	0	0
**R.m.s deviations**			
Bond lengths (Å)	0.016	0.003	0.003
Bond angles (°)	1.284	0.512	0.685
**Validation**			
MolProbity score	1.4	1.23	1.34
Clashscore	4.1	4.58	6.25
Rotamer outliers (%)	0.51	0.00	0.00
**Ramachandran plot**			
Favored (%)	96.72	98.82	99.50
Allowed (%)	3.13	1.18	0.50
Disallowed (%)	0.15	0.00	0.00
**Model vs. Data**			
CC (mask)	0.66	0.60	0.83
CC (box)	0.84	0.84	0.92
CC (volume)	0.66	0.57	0.80
CC (peaks)	0.61	0.49	0.75
CC (main chain)	0.67	0.73	0.89
CC (side chain)	0.71	0.78	0.91

Table S2: Cryo-EM data collection, refinement, and validation statistics for condensin II-ADP·BeF_3.

	Condensin II-ADP·BeF_3
**Data collection and processing**	
Microscope	TFS Glacios G2
Magnification	100,000
Voltage (kV)	200
Camera	Falcon 4i
Energy filter name	TFS Selectris
Energy filter slit width	15 eV
Automation software	EPU
Electron exposure ($e^-/Å^2$)	50
Defocus range (μm)	−1.2 to −2.4
Pixel size (Å)	1.18
Map sharpening B factor ($Å^2$)	−872.2
Map resolution (Å)	7.2
FSC threshold	0.143
EMDB entry	76416
Initial particle number	3,139,122
Final particle number	93,590
**Model Refinement**	
Model resolution (Å)	masked: 8.8 (7.5); unmasked: 8.9 (7.6)
FSC threshold	0.5 (0.143)
PDB entry	12GD
**Model composition**	
Non-hydrogen atoms	30,062
Protein residues	3,741
Nucleotides	0
Ligands	0
**R.m.s deviations**	
Bond lengths (Å)	0.012
Bond angles (°)	1.823
**Validation**	
MolProbity score	1.41
Clashscore	3.30
Rotamer outliers (%)	0.68
**Ramachandran plot**	
Favored (%)	95.77
Allowed (%)	4.09
Disallowed (%)	0.13
**Model vs. Data**	
CC (mask)	0.57
CC (box)	0.90
CC (volume)	0.49
CC (peaks)	0.49
CC (main chain)	0.79
CC (side chain)	0.81

Supplementary Files

This is a list of supplementary files associated with this preprint. Click to download.

- s1.mov
